# The origins of the full-field flash electroretinogram b-wave

**DOI:** 10.3389/fnmol.2023.1153934

**Published:** 2023-07-03

**Authors:** Yashvi Bhatt, David M. Hunt, Livia S. Carvalho

**Affiliations:** ^1^Centre for Ophthalmology and Visual Science, The University of Western Australia, Perth, WA, Australia; ^2^Lions Eye Institute Ltd., Nedlands, WA, Australia

**Keywords:** electroretinogram, b-wave, a-wave, bipolar cells, Müller glia cells, potassium ions

## Abstract

The electroretinogram (ERG) measures the electrical activity of retinal neurons and glial cells in response to a light stimulus. Amongst other techniques, clinicians utilize the ERG to diagnose various eye diseases, including inherited conditions such as cone-rod dystrophy, rod-cone dystrophy, retinitis pigmentosa and Usher syndrome, and to assess overall retinal health. An ERG measures the scotopic and photopic systems separately and mainly consists of an a-wave and a b-wave. The other major components of the dark-adapted ERG response include the oscillatory potentials, c-wave, and d-wave. The dark-adapted a-wave is the initial corneal negative wave that arises from the outer segments of the rod and cone photoreceptors hyperpolarizing in response to a light stimulus. This is followed by the slower, positive, and prolonged b-wave, whose origins remain elusive. Despite a large body of work, there remains controversy around the mechanisms involved in the generation of the b-wave. Several hypotheses attribute the origins of the b-wave to bipolar or Müller glial cells or a dual contribution from both cell types. This review will discuss the current hypothesis for the cellular origins of the dark-adapted ERG, with a focus on the b-wave.

## Introduction

First discovered in 1865 by Holmgren, the electroretinogram (ERG) measures the retina’s electrical activity in response to a light stimulus (Brown, 1968; Vinberg et al., 2014). It can evaluate the function of retinal cells within the photopic (cone-mediated) and the scotopic (rod-mediated) visual systems. The ERG is used in scientific research and clinical ophthalmology to study various eye diseases such as cone-rod dystrophy, rod-cone dystrophy, retinitis pigmentosa or diabetic retinopathy (Camuglia et al., 2011; Takao et al., 2017). In scientific research, *in vivo* and *ex vivo* ERG techniques are usually employed to explore the cellular and physiological origins of the ERG components, which in turn can help our understanding of visual processing and mechanisms that might lead to retinal disorders (Gundogan et al., 2011).

**Figure 1:**
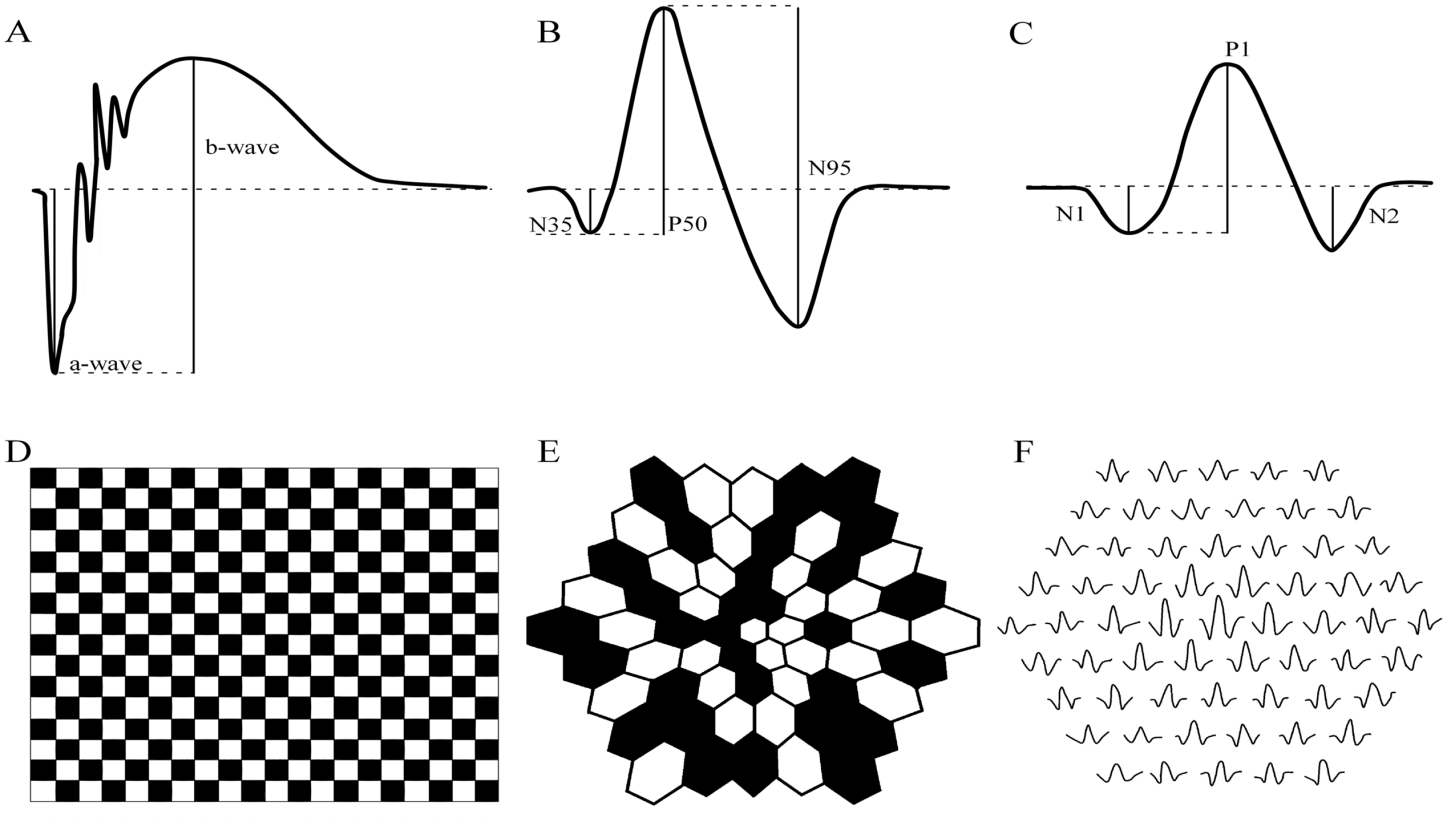
Schematic of the waveform of the three main types of Electroretinography (ERG) and their stimuli pattern. **(A–C)** Example traces of human responses and their respective amplitude peaks for quantification of the **(A)** full-field flash electroretinography (ffERG), **(B)** pattern electroretinography (PERG) and **(C)** multifocal electroretinography (mfERG). The way amplitude is measured for each waveform is represented by the solid black lines. **(D)** The contrast-reversing checkerboard stimuli of PERG and the **(E)** 61 hexagon stimuli for mfERG. **(F)** A representative trace array of 61 individual mfERG waveforms.

In the clinical setting and within the standard testing defined by the International Society for Clinical Electrophysiology of Vision (ISCEV), an ERG is utilized as a non-invasive measure of retinal function. Three main types of ERG testing are used to evaluate the health of the retina: full-field flash electroretinography (ffERG; Figure 1A), pattern electroretinography (PERG; Figure 1B), and multifocal electroretinography (mfERG; Figure 1C). Diagnostic for diseases of the retina and visual pathway, the ffERG is the mass electrical response to varying flash stimuli ranging from 0.01 to 10 cd·s·m^−2^ in the dark-adapted standard tests and in while the light-adapted standard tests the 3 cd·s·m^−2^ and the 30 Hz flicker stimuli are used (Robson et al., 2022). In contrast to ffERG, the PERG is more focused and records the retinal response from a specific area stimulated by a contrast-reversing checkerboard or grating stimulus (Figure 1D). The PERG usually uses a localized macular stimulus to assess the health and function of the macula in humans and the central retina in animals without maculae, specifically the photoreceptor cells and retinal ganglion cells (to assess optic nerve dysfunction), but a larger field stimulus could also be used (Holder et al., 2003; Lenassi et al., 2009; Bach et al., 2013). However, the ffERG and PERG fall short in their ability to simultaneously measure the retina’s electrical activity in multiple locations. This is resolved through a mfERG which can derive the electrical responses from multiple areas of the retina. The stimulus presented in mfERG is an array of 61 or 103 hexagons that flicker between black and white states in a pseudo-random sequence (Figure 1E). The responses obtained from mfERG are represented in trace arrays (field view) with either 61 or 103 elements, and this helps to assess the function of cone-photoreceptors and bipolar cells (Figure 1F; Hood, 2000; Hoffmann et al., 2021). As the most widely used test, this review will focus on the ffERG and the cellular origins of its different components, paying close attention to the origins of the dark-adapted b-wave.

### The dark-adapted a-wave

**Figure 2:**
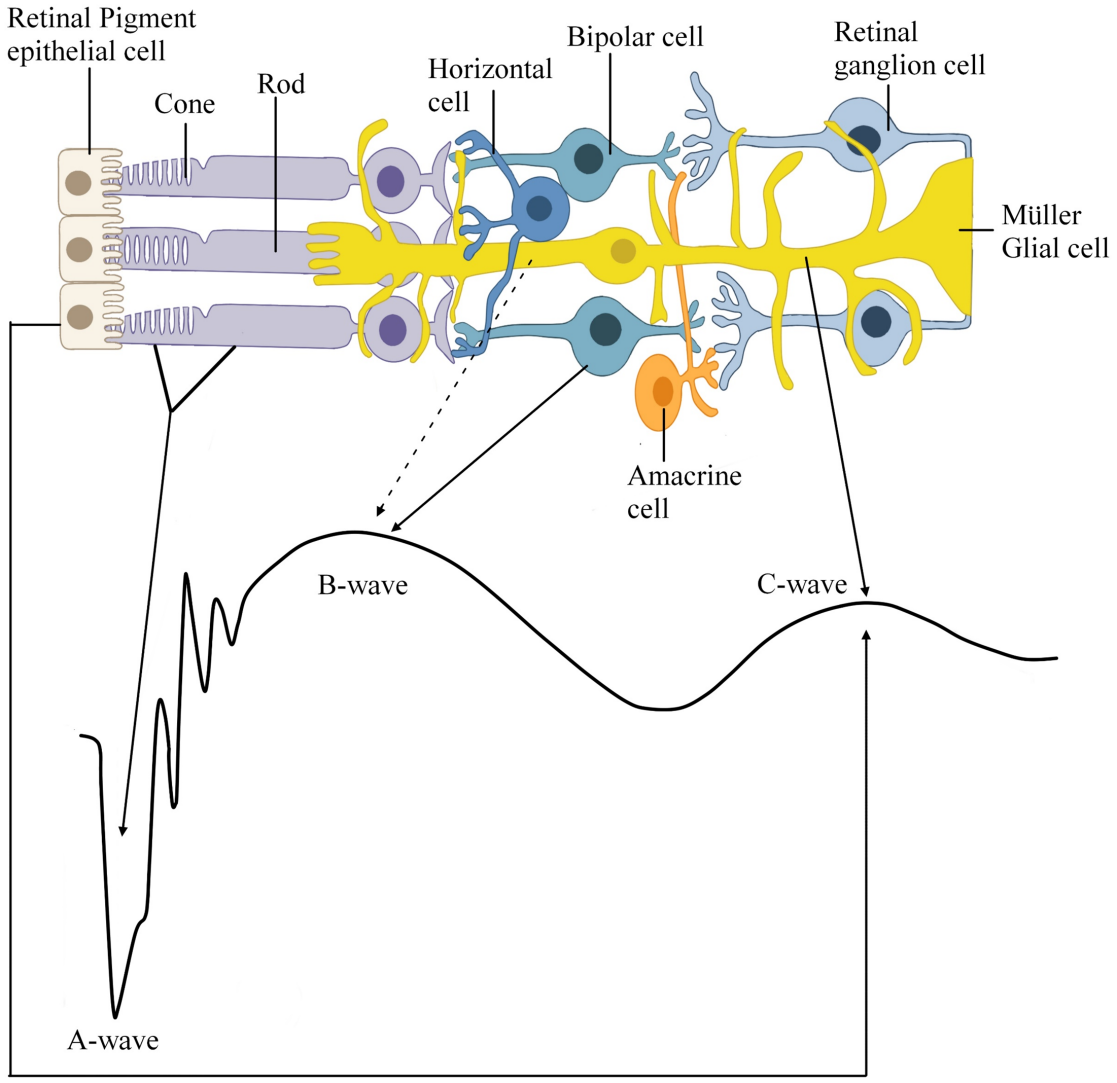
Schematic representation of the retina depicting which cell type contributes to which part of the *in vivo* scotopic electroretinogram (ERG) response recorded from a wildtype mouse (C57BL6/J). This recording was conducted via the Celeris DiagnosysLLC with a filter configuration of for the a- and b-wave the light intensity was 10 cd.s/m^2^ with a low filter frequency cut-off of 0.125 Hz and high filter frequency cut off of 300 Hz and for the c-wave the light intensity 150 cd.s/m^2^ was low filter frequency cut-off of 0.125 Hz and high filter frequency cut off of 100 Hz. Solid black lines represent a well-supported hypothesis, and the dashed black lines represent the possible links between the retinal cells and waveform.

Einthoven and Jolly (1908) divided the scotopic ERG into three components: (1) the initial negative deflection as the a-wave, (2) the first positive summit as the b-wave, and (3) the second positive summit as the c-wave (Figure 2). This work was then built upon by many others, but most remarkably by Granit (1933), who categorized the three components of the ERG by their order of disappearance as the level of anesthesia deepened. He labeled them as waves PI, PII and PIII. Though the origins of these components were not yet known, he suggested that the leading edge of the negative wave was the PIII and that the b-wave was composed of the PII and PIII, while the slow c-wave is the summation of PI and PIII. It was not until 1961 that a study by Brown and Wiesel (1961) recorded the ERGs of anaesthetized cats using micropipette electrodes and established that the scotopic a-wave is a response from retinal photoreceptors, specifically from rod outer segments. This study employed xylocaine to probe the origins of the a- and b-waves and found that the b-wave is eliminated through its use but that the amplitude of the a-wave increases, further supporting Granit’s (1933) idea that the b-wave is composed of PII and PIII. To Brown, this increase in a-wave amplitude and the absence of a b-wave suggested that the amplitude of the negative a-wave is interrupted by the rise of the positive b-wave, which also supports Granit’s assertion that the decline in PIII is interrupted by the rise in PII in response to long and dim flashes. Early studies by Hagins et al. (1970) and Penn and Hagins (1969) recording from the rat retinae were able to confirm a circulating current flowing extracellularly out of the rod outer segments and back into the cell at the inner segment region. This led to the theory that the a-wave was developed across the photoreceptor layer and originated in the rod’ outer and inner segments due to the similarity in the waveform of rod photocurrent and the a-wave (Brown, 1968; Penn and Hagins, 1969; Hagins et al., 1970).

In a later study, Hood and Birch (1990) fitted the leading edge of the rod-driven a-wave from individuals with normal vision and those with congenital stationary night blindness (CSNB), where rod vision is compromised, to a quantitative model of the ERG. This established that the amplitude of the leading edge of the a-wave is the sum of rod photoreceptor activity (Figure 2). This statement was then further supported by Lamb and Pugh (1992), who created a quantitative model describing the rising phase of the rod response based on the phototransduction cascade and found that the time course of the a-wave was similar to the suppressing circulating rod current. However, it is important to note that both these studies worked under the assumption that the rod outer segment current has the same waveform as the a-wave and that deviations in the ERG waveform just before or after the peak amplitude are due to the b-wave intrusion. While this model fits the a-wave generated in response to weak stimuli Robson and Frishman (2014) employed a slightly altered computational model with a better fit to the leading edge, the trough and the recovery toward the baseline, which posited that if the a-wave is generated via a strong stimulus and the peak is earlier than 12 ms, it is not just the sum of outer segment photocurrents but is also influenced by the transient capacitive current in the outer nuclear layer (ONL), specifically in the inner segment and axons. This inward current at the inner segments had already been shown to be driven by K^+^ influx by Matsuura et al. (1978) and Oakley et al. (1979) and is now known to be mediated by the Na^+^/K^+^ pump and closure of the K^+^ voltage-gated channels (Kv channels) present in the inner segments (Gayet-Primo et al., 2018; Fortenbach et al., 2021). Following on from this, recent studies have shown that in mouse models where the voltage-gated potassium channel (Kv) co-partner subunits Kv8.2 and Kv2.1 are knocked out, both show a reduction in the scotopic a-wave, but more importantly, a change in the shape of the a-wave trough described as the loss of the small negative peak that follows the initial negative peak in wildtype (WT) mice (Hart et al., 2019; Jiang et al., 2021; Inamdar et al., 2022). It has been reported that the Kv8.2 and the Kv2.1 subunits are found in rod and cone inner segments (Gayet-Primo et al., 2018). This change in the shape of the a-wave trough and the lack of Kv8.2/Kv2.1 channels from rod inner segments further support the idea that currents within the inner segments play a role in determining the a-wave. Somewhat contrary to this line of thought, Dang et al. (2011) found that in the rat retina, the use of CNQX (6-cyano-7-nitroquinoxaline-2,3-dione) to inhibit AMPA/KA (α-amino-3-hydroxy-5-methyl-4-isoxazole propionic acid and kainite sensitive) receptors, stops glutamatergic neurotransmission, resulting in a decrease in a-wave amplitude, as well as a change in the a-wave trough. This study suggested that the reduction in amplitude could be attributed to decreased receptor output, loss of ionotropic sensitive corneal negative responses, or the inhibition of AMPA/KA sensitive elements within the outer retina. Furthermore, Robson and Frishman (2014) suggested that the recovery of the a-wave to baseline in the absence of a b-wave response arises from the capacitive currents in rod inner segments and axons. However, it is essential to note that this does not contradict the fact that in the presence of the b-wave, the recovery phase of the a-wave to baseline is always contaminated by the response of the b-wave (Granit, 1933). Thus, exploring the post-receptoral contributions to the a-wave in mouse models, such as the no b-wave mutant mouse (*nob* mice).

### The dark-adapted b-wave

**Figure 3:**
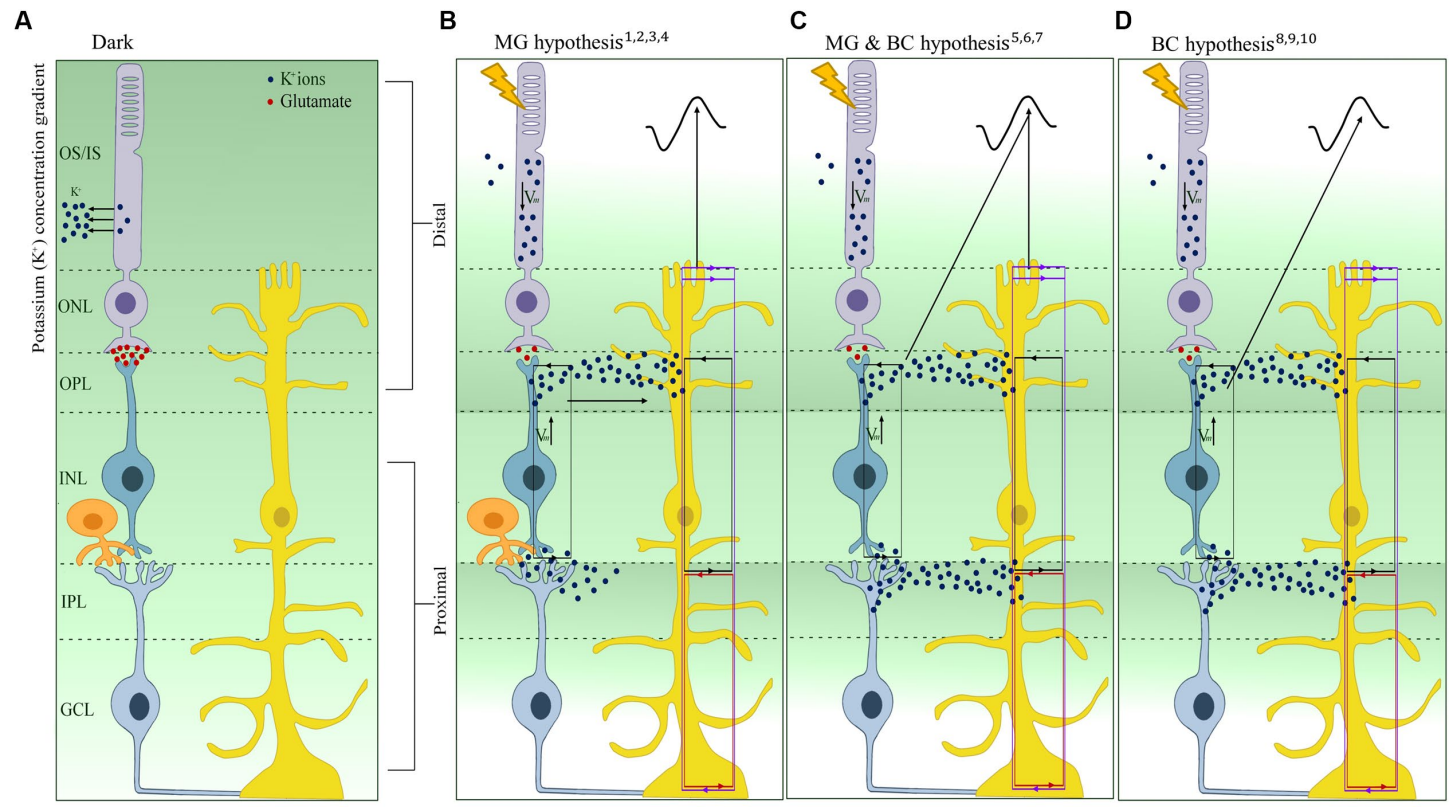
Schematic representation and overview of the three hypotheses linked to the b-wave origin after light activation. **(A)** Representation of the extracellular retinal potassium (K^+^) gradient in the dark. **(B)** Müller glia (MG) (or K^+^) hypothesis depicting the increase of K^+^ in the distal retina contributing to the change in extracellular K^+^ leading to the generation of the b-wave. **(C)** MG & bipolar cell (MG-BC) hypothesis depicting the change in membrane potential of BC and the change in extracellular K^+^ via K^+^ siphoning in the MG contribute to the generation of the b-wave. **(D)** BC hypothesis shows that the change in membrane potential of the BC alone contributes to the generation of the b-wave. Purple lines, source/sink for the SlowPIII; Red line, source/sink for the M-wave; black line, source/sink for the b-wave; *Vm*, change in membrane potential; OS, Outer segment; IS, Inner Segment; ONL, Outer Nuclear Layer; OPL, Outer Plexiform Layer; INL, Inner Nuclear Layer; IPL, Inner Plexiform Layer; GCL, Ganglion Cell Layer; Blue dots, K^+^ ions; Red dots, Glutamate. The references are ^1^Faber (1969), ^2^Dick and Miller (1978), ^3^Dick et al. (1985), ^4^Wen and Oakley (1990), ^5^Heckenlively and Arden (2006). ^6^Fujimoto and Tomita (1981), ^7^Newman and Odette (1984), ^8^Stockton and Slaughter (1989), ^9^Karwoski and Xu (1999), ^10^Gurevich and Slaughter (1993), ^11^Tian and Slaughter (1995), and ^12^Kofuji et al. (2000).

According to the current ISCEV (2022) standard, the b-wave response is driven: primarily by the rod-driven On-bipolar cells (BC). Granit (1933) first suggested that the b-wave is composed of the PII and PIII waves, and this theory has persisted, as ISCEV guidelines suggest that the b-wave is measured from the a-wave trough to the peak of the b-wave (Figure 1A). This section will review the three leading schools of thought for the origins of the b-wave: that the b-wave arises in either BC cells, Müller glial (MG) cells or both. For this section of the review, the literature mentioned has been summarized in Table 1 and consists of species, preparation and techniques used to record data. It is important to note that, contrary to anatomical terms of location, in this review, the distal retina refers to the outer retina, which spans from the outer plexiform to the retinal pigment epithelium layer and is closest to photoreceptors, whereas the proximal retina refers to the inner retina, which spans from the nerve fiber layer to the inner nuclear layer (Figure 3A; Vinekar et al., 2015; Pasmanter and Petersen-Jones, 2020). Therefore, light-induced responses occur from the distal to the proximal retina, beginning at the photoreceptors and ultimately sending neural responses from ganglion cells to the visual cortex (Pasmanter and Petersen-Jones, 2020).

**Table 1:** A summary of the studies mentioned within this review that comment on the origin of the b-wave.

Animal model	Preparation	Technique	References
Albino rabbits	Opened and closed-eye	Microelectrode with indifferent electrode behind the eye.	Faber (1969)
Mudpuppy (*Necturus maculosus*)	Eyecup	Microelectrode	Miller and Dowling (1970)
Rabbit	Eyecup	Double-barrelled K^+^- selective microelectrodes	Dick and Miller (1985)
Mudpuppy (*Necturus maculosus*)	Eyecup	Double-barrelled K^+^-selective microelectrodes	Dick and Miller (1978)
Toad (*Bufo marinus*)	Isolated retina	Double-barrelled K^+^- selective microelectrodes	Oakley (1975)
Toad (*Bufo marinus*)	Isolated retina	Double-barrelled K^+^- selective microelectrodes	Wen and Oakley (1990)
Bullfrog (*Rana catesbeiana*)	Eyecup and the isolated retina	Semi-micropipette (One filled with normal Ringer’s and the other with a high K^+^Ringer’s)	Fujimoto and Tomita (1981)
Mudpuppy (*Necturus maculosus*)	Eyecup	Double-barreled micropipettes and micropipettes filled with 3 M potassium acetate with a indifferent electrode behind the eye.	Karowski and Proenza (1977)
Bullfrog (*Rana catesbeiana*)	Eyecup and the isolated retina	Semi-micropipette (One filled with normal Ringer’s and the other with a high K^+^ Ringer’s)	Yanagida and Tomita (1982)
Mudpuppy (*Necturus maculosus*) and Tiger salamander (*Ambystoma tigrinium*)	Eyecup	Potassium ion-selective microelectrodes	Stockton and Slaughter (1989)
Skate (*Raja erinacea or R. oscellata*)	Small pieces of eyecup	Double-barrel micropipette fashioned from theta tubing.	Kline et al. (1978)
Mudpuppy (*Necturus maculosus*) and Frog (*Rana pipiens and R. p. berlandieri*)	Eyecup and retinal slice	Double-barrelled K^+^ selective microelectrodes	Karwoski et al. (1985)
Frog (*Rana pipiens*)	Eyecup	Ringer-filled micropipettes	Newman (1980)
Frog (*Rana pipiens*)	Eyecup	Double-barreled pipette made from theta tubing.	Xu and Karwoski (1994)
Rabbits (New Zealand White)	Eyecup	Double-barreled pipette made from theta tubing.	Karwoski and Xu (1999)
Tiger Salamandars (*Ambystoma tigrinum*, aquatic stage), Guinea pigs, dutch belted rabbits, pigmented mice (C57BL), cats, and one owl monkey (*Aotus trivirgatus*).	Dissociated cells	Recording (suction) electrodes were filled with 156 mM KCL and 125 mM KCL for mammals and salamanders, respectively.	Newman (1987)
Tiger Salamandars (*Ambystoma tigrinum*)	Eyecup	Ringer’s filled glass electrode.	Gurevich and Slaughter (1993)
Tiger Salamandars (*Ambystoma tigrinum*)	Eyecup	Low resistance electrode.	Tian and Slaughter (1995)
Cat	Intact cat eye (*in vivo*)	Intraretinal recording was done via double-barrelled K^+^ selective microelectrodes and vitreal ERG was recorded via chlorided silver wire in the vitreous humor and a reference behind the eye.	Frishman and Steinberg (1989a,b)
Cats and macaques	*In vivo*	In cats, the ERG is recorded between an intravitreal silver wire electrode and another behind the eye. In macaques, the ERG is recorded differentially from DTL fibers sandwiched between the cornea and a contact lens.	Robson and Frishman (1998)
Mice (C57BL/6J)	Isolated retina (*ex vivo*)	Double-barreled K^+^-selective microelectrodes	Dmitriev et al. (2021)
Pigmented Dutch rabbits	*In vivo*	Recorded with non- polarizing Ag-AgCI amalgam electrode pellets (in the vitreous and under the lid).	Hu and Marmor (1984)
Albino rabbits	*In vivo*	ERG responses were recorded simultaneously from both eyes with corneal electrodes.	Lei and Perlman (1999)
Albino rats (Sprague–Dawley)	Isolated retina (*ex vivo*)	Recordings were made between an Ag–AgCl macro-electrode built in the perfusion system chamber.	Green and Kapousta-Bruneau (1999)
Neotenous tiger salamander	Eyecup	K^+^-sensitive microelectrodes	Coleman et al. (1987)
Mice	Eyecup	ERG recorded from eye cup in a perfusion system chamber.	Kofuji et al. (2000)

#### The Müller glia-bipolar cells b-wave hypothesis

The link between MG and the b-wave was first made in 1969 by Faber, who, within the rabbit retina, suggested that the best fit for the generation of the b-wave is a depolarization of MG in the outer plexiform layer (OPL). This is due to the fact that Faber reported the source of the b-wave to lie proximally and distally to the OPL while the sink is located at the OPL. This was subsequently supported by the work of Miller and Dowling (1970) using mudpuppy (*Necturus maculosus*) retina; they suggested that the changes in K^+^ concentration due to the retinal neurons’ activation led to the depolarization of MG, which then leads to radial current flow that is recorded as the b-wave. This hypothesis was based on the similarity of the waveform the latency and intensity-response curve between the ERG and the MG responses. The accumulation of these observations forms the basis of the MG cell hypothesis, also known as the K^+^ hypothesis (Figure 3B). Similarly, within the rabbit retina, Dick and Miller (1985) observed two extracellular K^+^ increases at the onset of light (proximal and distal). The proximal K^+^ increase was attributed to ganglion and amacrine cells, and the distal extracellular K^+^ increase was attributed to the ON-BC depolarizing (Dick and Miller, 1978; Dick et al., 1985). They also observed a decrease in K^+^ ions in the distal retina, which was localized to the rod inner segments (Oakley, 1975). Based on the observation that light-induced depolarization of ON-BC causes an increase in K^+^, leading to the depolarization of MG, Dick and Miller (1985) suggested that it is this that is generates the radial current responsible for the b-wave. In 1990, using the then-new K^+^-selective microelectrodes on the isolated retina of the toad (*Bufo marinus*), Wen and Oakley (1990) found that the distal light-evoked K^+^ increase is large and fast enough to depolarize the MG in order to generate the b-wave. Additionally, when applying Ba^2+^, they observed a 65% decrease in the amplitude of the MG depolarization and the b-wave while only observing a < 10% decrease in the distal K^+^ levels. As Ba^2+^ blocks the K^+^ channels of the MG, the lack of uptake of K^+^ ions from the depolarization of the ON-BC results in a decrease in MG depolarization, thus not generating the b-wave (Wen and Oakley, 1990).

One of the points of support for the MG hypothesis was the similarity between the MG waveform and the b-wave, thereby linking the depolarization of the MG to the small distal increase in K^+^ in the OPL (Miller and Dowling, 1970; Dick and Miller, 1978). However, in the following years, this was contradicted by several studies; for example, Fujimoto and Tomita (1981) found that an injection of K^+^ within the frog retina led to a decrease in MG transretinal potential when injected in the distal retina compared to the proximal. This links, for the first time, the proximal retinal increase of K^+^ to MG depolarization, which was also confirmed by Karowski and Proenza (1977). While the MG hypothesis was still dominant, an in-depth profile analysis conducted by Yanagida and Tomita (1982) found that the b-wave reaches its peak at the outer plexiform layer, while the light-induced K^+^ increase peak is more proximal to the OPL. They posited that the difference in the light-induced peak of the K^+^ increase and the b-wave could not solely be the due to the MG but also to ON-BCs depolarizing in response to light via Na^+^ influx resulting in a current sink at the OPL and the K^+^ efflux. This latter efflux resulted in an increase in extracellular K^+^, suggesting for the first time that both MG and ON-BCs are needed for the generation of the b-wave (Figure 3C; Yanagida and Tomita, 1982). This was supported by Stockton and Slaughter (1989), who found that both distal and proximal increases of K^+^ are due to ON-BC depolarization, as the application of APB (2-amino-4-phosphonobutyrate), an ON-bipolar cell blocker, eliminated both K^+^ increases. Additionally, Frishman and Steinberg (1989b) suggest the increase in proximal K^+^ could also be from rod-driven amacrine and/or ganglion cells.

Contrary to these findings, and in line with the MG hypothesis, a computational model of the b-wave by Newman and Odette (1984) showed that if the distal K^+^ increase is as large as the proximal increase (based on the findings of Kline et al., 1978; Dick and Miller, 1985), then the retinal depth profile should peak at the OPL, similar to the b-wave. They also attributed the small distal K^+^ increase identified by some studies to the damage caused by the commonly used K^+^-selective microelectrode techniques used to measure changes in K^+^ concentration. Furthermore, unlike previous work, they posited that the proximal K^+^ increase depolarizes the MG to generate the b-wave, which could be possible, given that the MG cell spans the whole neuroretina. The increase in K^+^ at the OPL and IPL (inner plexiform layer) generates influxes into the MG, balanced by the outward currents at the OPL and IPL. An ILM (inner limiting membrane) source and IPL sink generate the M-wave, and the Slow PIII is generated by the OPL/OLM (outer limiting membrane source) and IPL sink, while the b-wave is said to be generated by an IPL source and OPL sink (Figure 3). Therefore, it is not the passive currents through the MG that generates the b-wave but the change in K^+^. It is this that generates transretinal currents and is called the b-wave (Newman, 1980; Karwoski et al., 1985; Xu and Karwoski, 1994; Karwoski and Xu, 1999).

Furthermore, when evaluating the b-wave origins, the work of Xu and Karwoski (1994) and Karwoski and Xu (1999) observed a depression (in frog retinas) and enhancement (in rabbit retinas) of the b-wave after the application of Ba^2+^. They observed that 60–70% of the b-wave directly generated from the ON-BCs was resistant to Ba^2+^, while 30–40% was Ba^2+^ sensitive, suggesting an MG origin (Xu and Karwoski, 1994). In contrast, the enhancement of the b-wave was attributed to the M-wave source and sink being ablated by Ba^2+^ application (Xu and Karwoski, 1994). It is important to note that the extent to which the MG plays a role in the generation of the b-wave might be species-dependent as K^+^ conductance distribution depends on whether the retinae are vascularized (the excess K^+^ is delivered to retinal capillaries via distal processes) or avascularized (excess K^+^ is transferred to vitreous humor via endfeet). Thus, suggesting that the current generated by the MG would differ between species due to the movement of the K^+^ (Newman, 1987).

#### The BC-only b-wave hypothesis

An alternate hypothesis suggests that light-induced depolarization of ON-BCs alone directly generates the b-wave. The early work of Dick and Miller (1978) in the mudpuppy retina showed that an application of a light stimulus increased K^+^ levels in both the proximal (at the level of post-BC) and distal (at the level of BC) retina. While the proximal increase in K^+^ is greater than the distal, it was found to be insignificant in the generation of the b-wave (Dick and Miller, 1978). This suggests that the increase in K^+^ at the level of the BC (distal retina) is solely responsible for the generation of the b-wave (Figure 3C). This concept was then further supported by two studies on the tiger salamander retina which observed that the b-wave and the ON-BC responses have similar waveforms (Gurevich and Slaughter, 1993; Tian and Slaughter, 1995). Furthermore, when APB, a selective blocker for BCs, is applied, it leads to the complete suppression of the b-wave, unlike the MG selective blocker Ba^2+^, which leads to a 45–65% suppression of the b-wave (Wen and Oakley, 1990; Gurevich and Slaughter, 1993; Xu and Karwoski, 1994). Within the MG-BC theory, the MG need the K^+^ extruded by the BC to depolarize and generate the transretinal currents that are recorded as the b-wave. Thus, it is important to realize that any effects on the b-wave by pharmacological agents that prevent the function of the BC do not rule out the possible contribution of the MG.

In support of the BC-only hypothesis, Hood and Birch (1996) used a computational model to remove the PIII component of human rod b-wave responses, suggesting that the remaining derived PII (which is the b-wave with rod response removed) is a better measure for BC activity when compared to the b-wave itself as they show an identical response. Their modeling also indicated that analyzing the full b-wave can be misleading as it underestimates the amplitude of BC activity by 15% and a delayed implicit time (Hood and Birch, 1996). This model runs under the assumption that the BCs directly generate the b-wave. It has been suggested that the P2 has a linear relationship between amplitude and stimuli strength (weak-moderate) such that the summed responses of BC are proportional to the number of BC activated (Robson and Frishman, 1995; Robson et al., 2004). Furthermore a similarity in timecourse between P2 and single cell current recording suggest a BC origin to the P2 (Robson et al., 2004). An alternative explanation for the origin of P2 is that it comprises two responses; a fast component that reflects the BC, which is responsible for the leading edge, and a slower component from the MG, which is responsible for the tail of the b-wave. Furthermore, the application of Ba^2+^ did not eliminate the P2 response but removed the slow, extended tail of the response within the cat retina (Frishman and Steinberg, 1989a; Frishman and Steinberg, 1989b; Robson and Frishman, 1998; Frishman and Wang, 2011). The BC-only theory is further supported by Dmitriev et al. (2021), who employed a relatively simple linear electrical computational model of MCs combined with experimental light-induced K^+^ changes in the mouse retina, which found that the MG potential is too small to have any significant contribution to the b-wave (Dmitriev et al., 2021). Furthermore, this study compared real ERGs of C57BL/6J mice to calculate MG responses with varying parameters to determine its role in the b-wave, concluding that MG does not contribute to the b-wave (Dmitriev et al., 2021). The BC-only theory is further supported by the Kir4.1 knockout mouse model, which knocks out the main inwardly rectifying K^+^ channels (Kir) in MG, which is thought to be responsible for the spatial buffering of extracellular K^+^ ions. Within this mouse model, no difference was observed in the b-wave of normal and mutant mice, while the slow PIII response, believed to be generated by K^+^ current flow through MG cells, was absent in the mutant mice and eliminated by application of Ba^2+^ in the wild type mice (Kofuji et al., 2000).

One of the main points of contention against the MG hypothesis is the persistence of the b-wave in the presence of Ba^2+^. Within the literature, the most common way to probe the function of the MG is through the application of Ba^2+^, which is an inorganic cation that non-specifically blocks the predominant channels of the MG, the K^+^ inward rectifying channels (Kir) (Alagem et al., 2001; Skatchkov et al., 2006). It is important to note however that the application of Ba^2+^ can result in responses that vary widely depending on the animal model and the mode of application; it can lead to the depression of the b-wave in *ex vivo* preparations of frog retina (Xu and Karwoski, 1994) and via an intravitreal injection into the rabbit eye (Hu and Marmor, 1984). The application of Ba^2+^ can also lead to the enhancement of the b-wave via intravitreal injection into the the rabbit eye (Lei and Perlman, 1999) or when perfused in an eyecup of albino rats (Green and Kapousta-Bruneau, 1999). Contrarily no observable effects are found when Ba^2+^ is perfused in the retina-eyecup of the tiger salamander (Coleman et al., 1987). The literature suggests that for the MG hypothesis to be accurate, the b-wave needs to be depressed in the presence of a Kir channel blocker, which attenuates the function of the MG. As this was not proven to be the case in the Kir4.1 KO mouse study (Kofuji et al., 2000), an alternative proposal would be that the enhancement of the b-wave after Ba^2+^ application could point to an inhibitory/modulatory contribution of the MG to the b-wave, allowing the b-wave to be within the normal range of function. This is potentially supported by supernormal b-wave responses seen in patients with *KCNV2* retinopathy, where the lack of functional voltage-gated K^+^ channels most likely cause a K^+^ imbalance in the retina (Wu et al., 2006).

An alternative explanation of the evidence proposed by Kofuji et al. (2000) is that BC might compensate for the loss of function from the MG, resulting in a b-wave that is seemingly “normal” thus masking the effects of MG loss. On the other hand, if MG does not play a role in generating the b-wave as seen in the Kir4.1 KO mice, then it poses another question: which retinal or glia cells, and which channels, is Ba^2+^ acting upon to either enhance or reduce the b-wave? Thus, this review demonstrates that historically, via *ex vivo* and *in vivo* experimentation in amphibian and mammalian animal models, the origins of the b-wave have been narrowed down to two main cells, MG and ON-BC. There is still some hesitation in the field toward completely dismissing the role of MG in the generation of the b-wave since its overall role in the retina is to maintain homeostasis, making its contribution, or lack thereof, difficult to study in isolation.

## Future directions and Conclusions

A full-flash electroretinogram is a functional tool that can help clinicians and researchers ascertain the physiology of the retinal cells in various retinopathies. It allows for insight into problems involving excitation and transmission of signals and ionic dysfunction in retinal cells. Understanding the components that form the ERG can aid researchers in establishing the outcomes of therapies via non-invasive methods. Furthermore, as more is understood about retinal diseases, ERG responses with specific changes in the waveform allow it to act as a non-invasive diagnostic tool, such as for *KCNV2* retinopathy and Juvenile X-linked retinoschisis (Sieving et al., 1999; Kutsuma et al., 2019). Additionally, ERGs can potentially be predictive in other complex retinal diseases, such as diabetic retinopathy and glaucoma severity, and also for neurological disorders, such as bipolar disorder and schizophrenia (Yonemura et al., 1962; Sarossy et al., 2021; Moreau et al., 2022; Peredo et al., 2022). In terms of retinal diseases, predictions such as these can allow for adequate time for gene therapy or pharmacological interventions to take place. This review provides a historical overview of the groundbreaking studies that explored and probed the cellular drivers of the a- and b-waves and the conflicting literature on their origins.

## Author contributions

All authors listed have made a substantial, direct, and intellectual contribution to the work and approved it for publication.

## Conflict of interest

The authors declare that the research was conducted in the absence of any commercial or financial relationships that could be construed as a potential conflict of interest.

## Publisher’s note

All claims expressed in this article are solely those of the authors and do not necessarily represent those of their affiliated organizations, or those of the publisher, the editors and the reviewers. Any product that may be evaluated in this article, or claim that may be made by its manufacturer, is not guaranteed or endorsed by the publisher.
